# Integrated Analysis of Rat Pulmonary Hypertension Datasets Identifies Candidate circRNA Networks in Hypoxia-Exposed Pulmonary Artery Tissue

**DOI:** 10.3390/ijms27188195

**Published:** 2026-09-15

**Authors:** Benjamin Atchison, Sean Byars, Priscilla R. Prestes, Mark Myers, Yutang Wang, Fadi J. Charchar

**Affiliations:** 1 Health Innovation and Transformation Centre (HITC), Institute of Innovation, Science and Sustainability (IISS), Federation University Australia, Ballarat, VIC 3350, Australia; benjaminatchison@students.federation.edu.au (B.A.); p.prestes@federation.edu.au (P.R.P.);; 2 Australian Centre for Blood Diseases, Monash University, Melbourne, VIC 3004, Australia; sean.byars@monash.edu; 3 Center for Active Living, Taylor’s University, Subang Jaya 47500, Selangor, Malaysia; 4 Department of Cardiovascular Sciences, University of Leicester, Leicester LE1 7RH, UK

**Keywords:** circular RNA, hypertension, rat, pulmonary artery, lung

## Abstract

Pulmonary hypertension (PH) is a progressive and life-threatening disease characterised by elevated pulmonary arterial pressure, vascular remodelling and right ventricular dysfunction. Emerging evidence implicates circular RNAs (circRNAs) in the regulation of vascular remodelling and inflammation; however, their contribution to PH remains poorly understood. Here, we performed an in silico analysis using a multi-tool circRNA discovery pipeline across three publicly available transcriptomic datasets derived from experimental rat models of PH: H_PRN (PRJNA809145; pulmonary artery tissue from normoxic and hypoxia-induced PH rats; 21% vs. 10% O_2_; *n* = 7), H_GSE (GSE159668; right lung tissue from normoxic and hypoxia-induced PH rats; 21% vs. 10% O_2_; *n* = 6) and M_PRN (PRJNA732522; total lung tissue from control and monocrotaline-induced PH rats; *n* = 6). CircRNAs were detected in all datasets; however, robust differentially expressed candidates were identified primarily in the hypoxia-exposed pulmonary artery dataset (H_PRN). In this dataset, three circRNAs—circCnot6l, circSmoc1 and circNtrk3—met the differential expression criteria (Benjamini-Hochberg adjusted *p*-value (FDR *q*) < 0.05, absolute log_2_ fold-change > 1). Downstream network analysis from unpaired samples prioritised circCnot6l and circNtrk3 as potential regulators of circRNA–microRNA (miRNA) and miRNA–messenger RNA axes involving miR-204-5p and miR-205, respectively. The predicted target networks were enriched for genes associated with key PH-related processes, including cell proliferation, adhesion, migration, angiogenesis and vascular development. Collectively, these findings identify circCnot6l and circNtrk3 as promising circRNA candidates involved in hypoxia-induced pulmonary artery remodelling and provide a foundation for future experimental studies investigating circRNA-mediated mechanisms underlying PH pathogenesis.

## 1. Introduction

Cardiovascular disease (CVD) remains the leading cause of morbidity and mortality worldwide [1], encompassing a broad range of disorders affecting the heart and blood vessels, including coronary artery disease, stroke, heart failure, and hypertension [2,3,4,5]. Among the major modifiable risk factors for CVD, hypertension is particularly important due to its high prevalence and its role in driving vascular dysfunction, end-organ damage, and adverse cardiovascular outcomes [6,7].

While systemic hypertension affects the systemic circulation, pulmonary hypertension (PH) is a distinct and progressive vascular disorder characterised by elevated pressure within the pulmonary circulation, with an estimated global prevalence of 2.28 per 100,000 individuals [8]. PH is associated with progressive pulmonary vascular remodelling, increased pulmonary vascular resistance and, ultimately, right ventricular dysfunction. As no curative treatment currently exists, further research into the molecular mechanisms underlying PH and the identification of potential therapeutic targets remains critically needed.

Circular RNAs (circRNAs) are a class of non-coding RNA generated through back-splicing of precursor messenger RNA (mRNA), whereby the 5′ and 3′ ends are joined to form a covalently closed loop [9]. Because they lack a polyadenylated tail, circRNAs are generally more stable than linear RNAs and resistant to exonuclease-mediated degradation. These properties, together with their tissue- and context-specific expression patterns, have led to growing interest in circRNAs as regulators of disease-associated transcriptional networks.

CircRNAs can influence gene regulation through several mechanisms. They may act as transcriptional regulators through interactions with RNA polymerase II, promoter regions or R-loop formation, and may also function indirectly as microRNA (miRNA) sponges, protein scaffolds or protein-binding decoys. In some contexts, circRNA may also undergo translation when they contain an open reading frame together with an internal ribosome entry site or N6-methyladenosine modification [10,11,12].

Despite growing evidence that circRNAs may contribute to PH pathobiology, their systematic identification remains technically challenging. Their unconventional sequence architecture, generally low expression levels and reliance on accurate back-splice junction detection mean that many circRNAs may be missed by single-tool approaches [13,14,15]. To address this, multi-tool computational pipelines have been developed to reduce false-positives and improve sensitivity and reproducibility compared with individual circRNA detection tools [16]. For instance, the in-development Nextflow nf-core/circrna pipeline integrates multiple circRNA detection tools and supports downstream miRNA target prediction and differential expression analysis [17]. Similarly, CirComPara2 runs multiple tools in parallel to improve back-splice detection and aggregates count data into a unified output, reducing redundancy [18].

In this study, we performed a multi-dataset analysis of publicly available rat PH transcriptomic datasets to identify candidate circRNAs and associated regulatory networks. Three datasets were analysed, representing hypoxia-exposed pulmonary artery tissue (PRJNA809145/H_PRN; Sprague-Dawley rats; 21% vs. 10% O_2_; *n* = 7), hypoxia-exposed lung tissue (GSE159668/H_GSE; Sprague-Dawley rats; 21% vs. 10% O_2_, *n* = 6) and monocrotaline-induced PH lung tissue (PRJNA732522/M_PRN; Wistar rats, *n* = 6) [19,20,21]. This design allowed us to screen for circRNA signals across related but biologically distinct PH contexts. Robust differentially expressed circRNA candidates were identified primarily in the hypoxia-exposed pulmonary artery dataset, leading us to prioritise circCnot6l and circNtrk3 for downstream miRNA, messenger RNA (mRNA), co-expression and pathway analyses.

## 2. Results

The analysis was structured as a staged prioritisation workflow. First, circRNAs were detected across three rat PH-related datasets using multiple circRNA discovery tools. Second, candidate circRNAs were prioritised based on differential expression and cross-tool support. Third, because false discovery rate (FDR)-significant circRNA candidates were identified primarily in H_PRN, the hypoxia-exposed pulmonary artery dataset, downstream miRNA, mRNA, co-expression and pathway analyses focused on H_PRN.

### 2.1. Multi-Dataset circRNA Screening and H_PRN-Focused Transcriptomic Prioritisation

Detection software revealed an abundance of individual circRNA hits from H_PRN (*n* = 12,713) and M_PRN (*n* = 13,241), with H_GSE showing the highest count overall (*n* = 19,340, Figure S1A). In total, 2841 circRNAs were common across all three datasets (Figure S1B). Detection profiles differed between circRNA quantification tools, with circRNA_finder identifying more circRNAs than CIRIquant, CIRI2 and find_circ across several comparisons (Figure S1C–E). However, broad circRNA detection did not translate into consistent differential expression across all datasets, so candidate prioritisation was based on differential expression together with cross-tool support.

In total, 718 circRNAs were nominally differentially expressed across the three datasets (Figure S2A, *p* < 0.05, absolute log_2_FC > 1), including candidates shared between datasets. However, only 31 nominally differentially expressed circRNAs were detected by more than one tool (Figure S2B), indicating limited cross-tool support for many candidates. The strongest differential circRNA signal was observed in H_PRN, whereas H_GSE and M_PRN contributed fewer candidates meeting the prioritisation criteria. Among the detection tools, CIRI2, CIRIquant and circRNA_finder showed the greatest support for candidate circRNAs, while find_circ showed more limited discovery in some datasets.

After applying the prioritisation criteria, (Figure S8), 10 circRNA candidates were retained: three FDR-significant differentially expressed circRNAs and seven nominally significant circRNAs (Table 1). All three FDR-significant candidates—circNtrk3_0005, circSmoc1_0009 and circCnot6l_0006—were identified in H_PRN, the hypoxia-exposed pulmonary artery dataset. The remaining nominally significant candidates were distributed across H_PRN and M_PRN. Generalised linear mixed modelling indicated concordant inter-tool support for the prioritised circRNAs, with circSmoc1_0009 and circNtrk3_0005 showing particularly strong cross-tool similarity.

CircNtrk3_0005 (circNtrk3) was differentially expressed across all four tools (Benjamini-Hochberg adjusted *p*-value (FDR *q*); CIRI2 = 1.60 × 10^−3^, circRNA_finder = 2.61 × 10^−4^, CIRIquant = 0.0371, find_circ = 1.52 × 10^−3^) and had the strongest overall statistical support among the candidate circRNAs. CircSmoc1_0009 (circSmoc1) was differentially expressed according to CIRI2 (FDR *q* = 0.002) and circRNA_finder (FDR *q* = 2.71 × 10^−7^) and nominally significant according to CIRIquant (*p* = 0.0005) and find_circ (*p* = 0.0005). CircCnot6l_0006 (circCnot6l) was differentially expressed according to CIRIquant (FDR *q* = 0.02) and nominally significant according to circRNA_finder (*p* = 0.005) and find_circ (*p* = 0.02). Because all three FDR-significant candidates were identified in H_PRN, subsequent miRNA and mRNA analyses were performed using the corresponding H_PRN transcriptomic data. circCnot6l and circNtrk3 were prioritised for further network analysis because their predicted miRNA targets were also differentially expressed in H_PRN.

**Figure 1:**
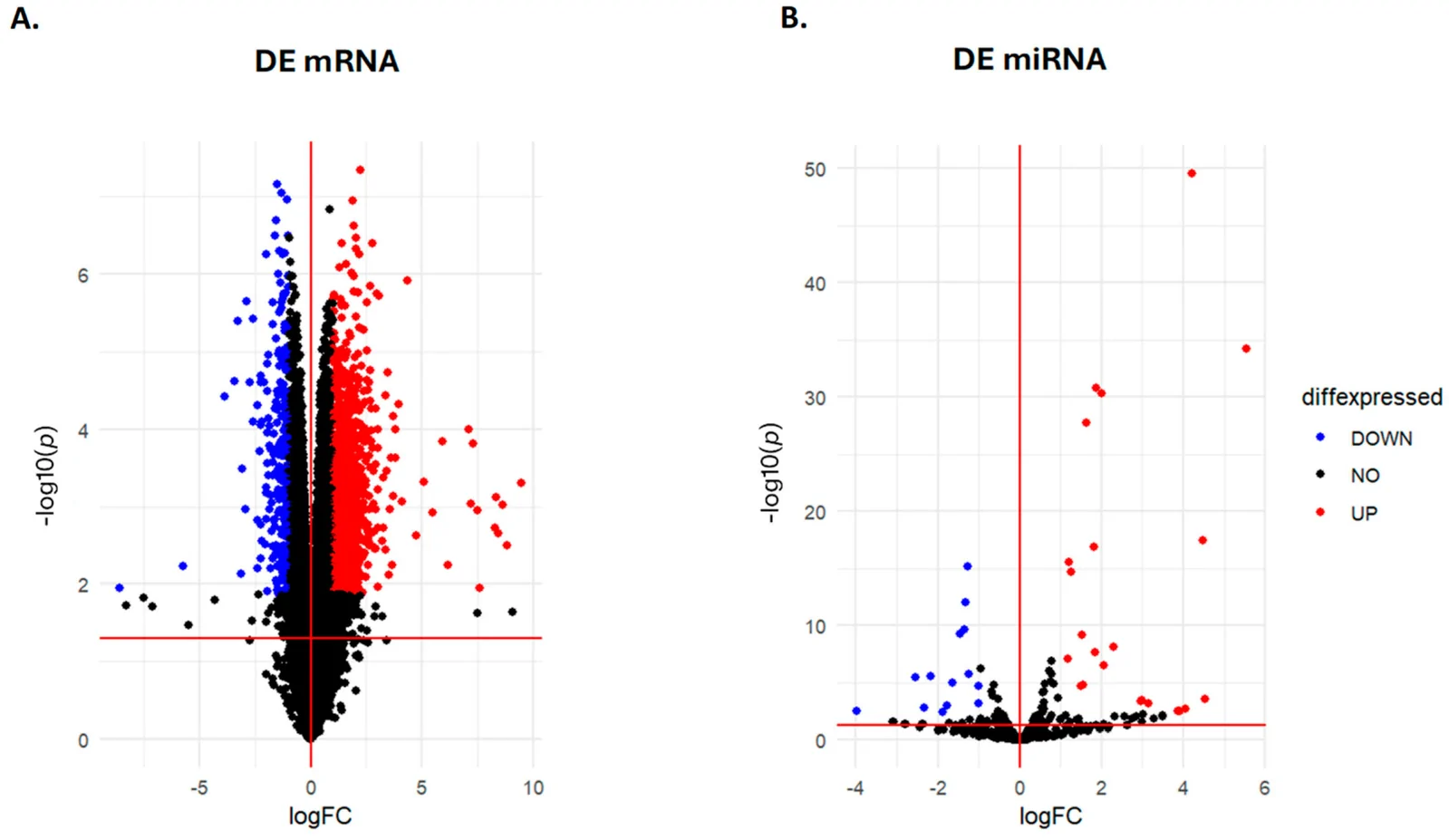
Volcano plot series representing log2 fold-change (log2FC, x-axis) against the negative base −10 logarithm of *p*-value (−log10(*p*), y-axis). (**A**) mRNA (messenger RNA; *n*(sample) = 7), (*n*(mRNA) = 28,109), (*n*(differentially expressed (DE) mRNA) = 1335). (**B**) miRNA (microRNA; *n*(sample) = 10), (*n*(miRNA) = 585, *n*(DE miRNA) = 37). Study, H_PRN (PRJNA809145, Total pulmonary artery tissue (TPAT), HPH vs. control). HPH, hypoxia-induced pulmonary hypertension; UP, significantly upregulated (Benjamini-Hochberg adjusted *p*-value (FDR *q*) < 0.05 and log2FC > 1). DOWN, significantly downregulated ((FDR *q*) < 0.05 and log2FC < −1). NO, not significantly DE. Plots generated in R. (Version 4.3.2) using ggplot2 (Version 3.5.1).

In H_PRN, 28,109 mRNA transcripts were quantified, of which 1335 were differentially expressed between hypoxia-exposed and control pulmonary artery tissue samples (Figure 1A and Table S4; 1062 upregulated and 273 downregulated). In the corresponding miRNA data, 585 mature miRNA sequences were identified, of which 37 were differentially expressed (Figure 1B; 23 upregulated and 14 downregulated).

### 2.2. Functional Enrichment of H_PRN Differentially Expressed Genes

Enrichment analysis of 2510 upregulated and 2040 downregulated statistically significant genes (Table S4, FDR *q* < 0.05) from the H_PRN dataset revealed a host of networks termed by gene ontology. Several biological processes were significantly upregulated, including positive regulation of cell migration, cell adhesion mediated by integrin, positive regulation of vasculature development and positive regulation of angiogenesis (Table S1, FDR *q* < 0.05). In addition, supramolecular fibre organisation, response to cytokine, actin filament organisation, signal transduction and anatomical structure development were found significantly downregulated (Table S2, *p* < 0.05).

### 2.3. Target Prediction and Network Construction

**Figure 2:**
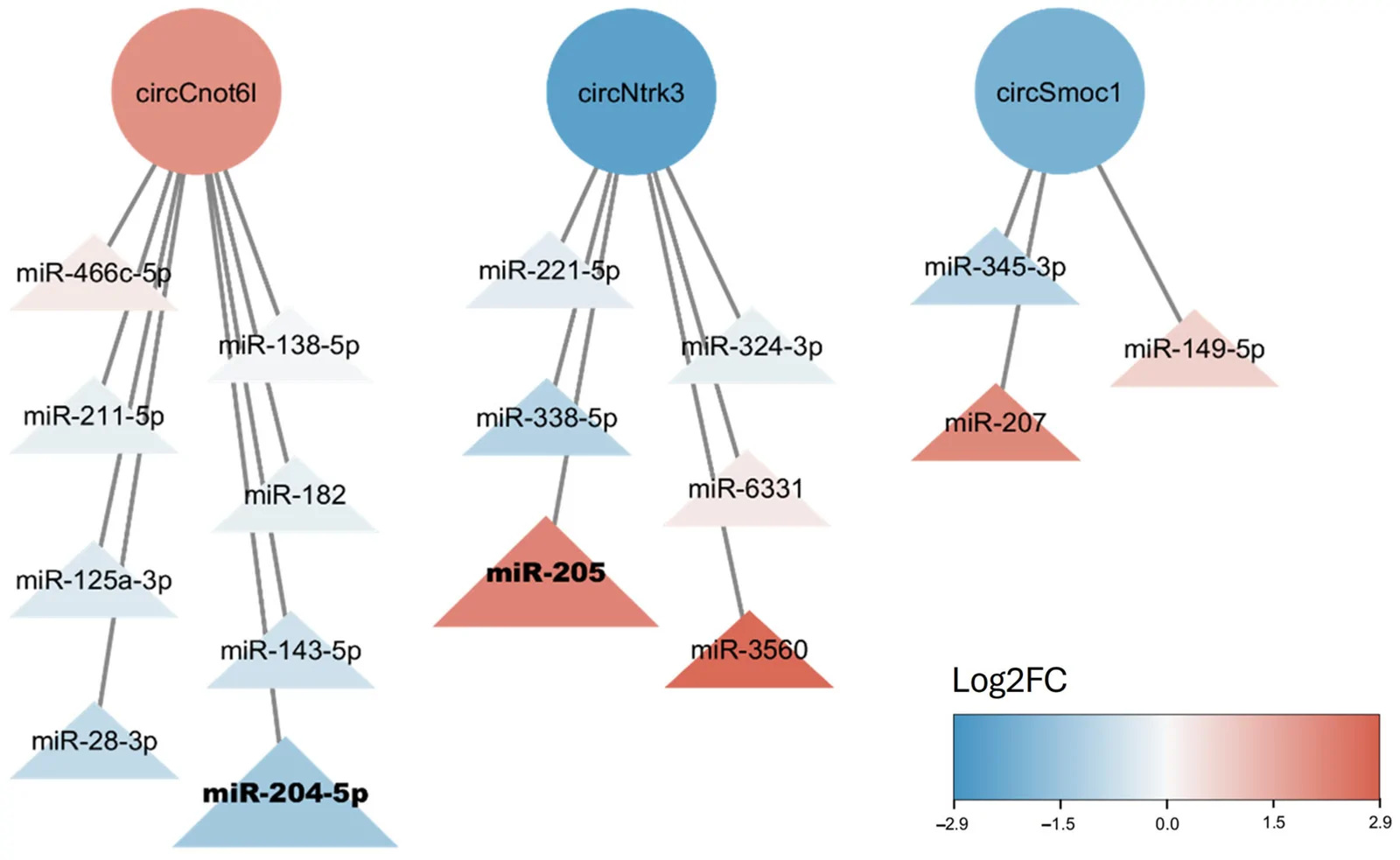
Predicted competitive endogenous RNA regulatory network for candidate circular RNAs (circRNAs). Bold/enlarged microRNA (miRNA) nodes are differentially expressed (FDR *q* < 0.05, log2FC > 0.95). log2FC, log2 fold-change (HPH phenotype vs. control); CircRNA, circular RNA; HPH, hypoxia-induced pulmonary hypertension. Expression data manipulated in R. environment (Version 4.3.2) and figure presented by Cytoscape (Version 3.10.2).

The three FDR significant H_PRN circRNA candidates—circCnot6l, circNtrk3 and circSmoc1—were assessed for predicted miRNA interactions. Across these candidates, 17 putative miRNA targets were identified (Figure 2). Integration with H_PRN miRNA differential expression data from unmatched samples identified miR-204-5p as differentially expressed (FDR *q* < 0.05, log2FC = −0.95) and a predicted target of circCnot6l and miR-205 as a differentially expressed (FDR *q* < 0.05, log2FC = 1.53) and predicted target of circNtrk3. No predicted circSmoc1-associated miRNA target met the differential expression criteria; so, circSmoc1 was not taken forward for downstream mRNA network analysis.

**Figure 3:**
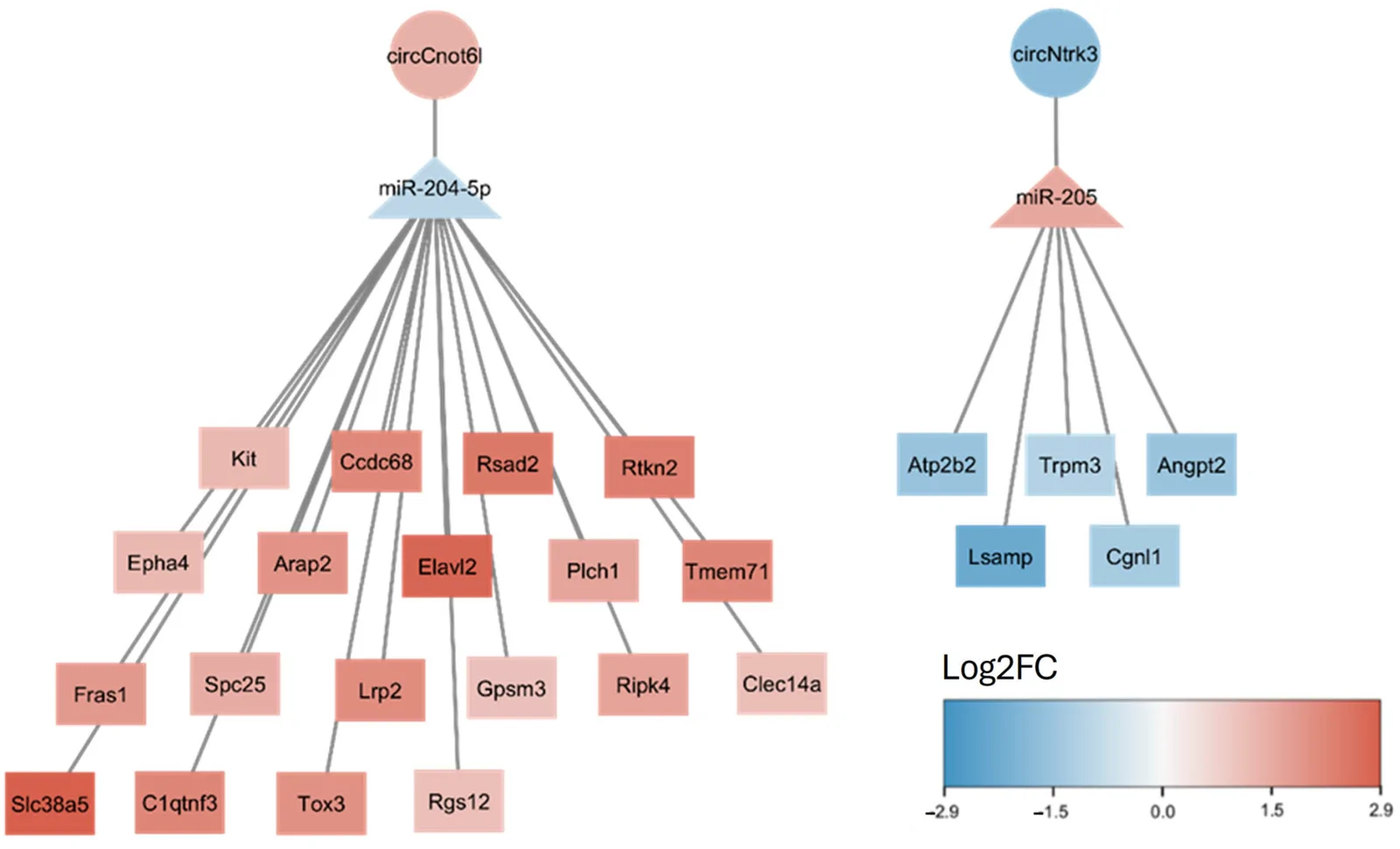
Full endogenous regulatory network (log2FC > 0.95, FDR *q* < 0.05) for circRNA and downstream differentially expressed targets. CircCnot6l (*n*(miRNA) = 1, *n*(mRNA) = 19), CircNtrk3 (*n*(miRNA) = 1, *n*(mRNA) = 5). CircRNA, circular RNA; miRNA/miR, microRNA; HPH, hypoxia-induced pulmonary hypertension; log2FC, Log2 fold-change (pulmonary hypertension phenotype vs. control). Downstream rectangular targets represent gene interactions. This network was generated using Cytoscape (Version 3.10.2). Abbreviations: *Angpt2*, Angiopoietin-2; *Arap2*, ArfGAP With RhoGAP Domain; *Atp2b2*, ATPase Plasma Membrane Ca2+ Transporting 2; *C1qtnf3*, C1q And TNF Related 3; *Ccdc68*, coiled-coil domain containing 68; *Cgnl1*, Cingulin Like 1; *Clec14a*, C-type lectin domain family 14 member A; *Elavl2*, Elav-like RNA-binding protein 2; *Epha4*, Ephrin type-A receptor 4; *Fras1*, Fraser extracellular matrix complex subunit 1; *Gpsm3*, G Protein Signalling Modulator 3; *Kit*, Proto oncogene c-KIT; *Lrp2*, low-density lipoprotein-related protein 2; *Lsamp*, Limbic System-Associated Membrane Protein; *Plch1*, Phospholipase C Eta 1; *Rgs12*, Regulator of G Protein Signalling 12; *Ripk4*, Receptor Interacting Serine/Threonine Kinase 4; *Rsad2*, Radical S-adenosyl methionine domain-containing protein 2; *Rtkn2*, Rhotekin 2; *Slc38a5*, Solute Carrier Family 38 Member 5; *Spc25*, SPC25 Component Of NDC80 Kinetochore Complex; *Tmem71*, Transmembrane Protein 71; *Tox3*, TOX High Mobility Group Box Family Member 3; *Trpm3*, Transient Receptor Potential Melastatin 3.

Target prediction identified 628 putative mRNA targets downstream of the circCnot6l/miR-204-5p and circNtrk3/miR-205 axes (Figure S4). After integration with H_PRN mRNA differential expression and filtering for inverse miRNA–mRNA expression patterns from unmatched samples, 24 differentially expressed mRNAs remained: 19 associated with the candidate circCnot6l/miR-204-5p axis and five associated with the candidate circNtrk3/miR-205 axis (Figure 3).

### 2.4. Co-Expression Analysis of Prioritised H_PRN circRNA–mRNA Candidate Networks

**Figure 4:**
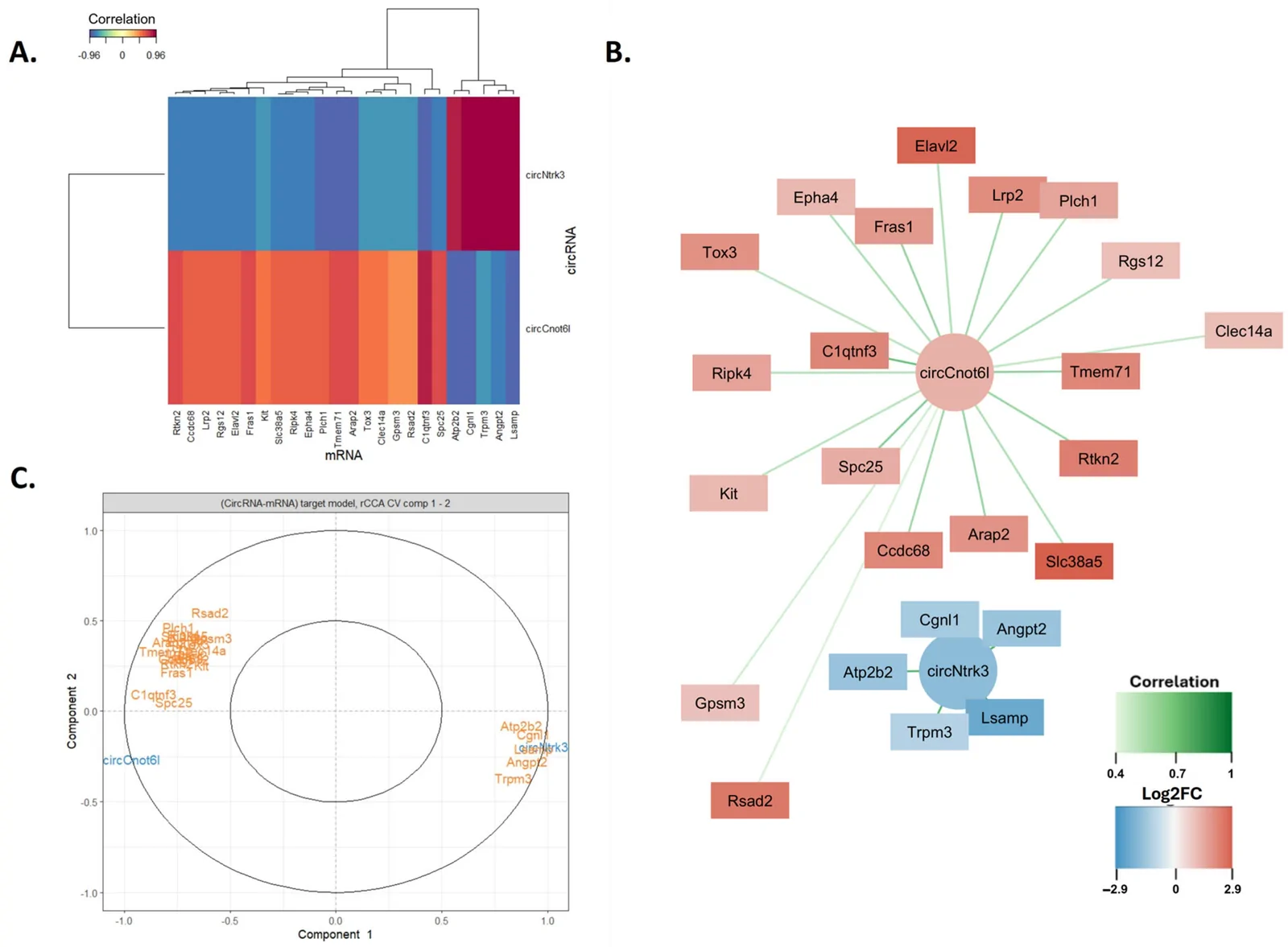
Correlational analysis between circRNA candidates (circCnot6l, circNtrk3) and their respective downstream targets. (**A**) Clustered heatmap. CircRNA (y-axis), messenger RNA (mRNA; x-axis). Correlation of “1” (red) infers complete relationship, correlation of “−1” infers opposite relationship and correlation of “0” indicates no relationship. (**B**) Covariate network of circCnot6l, circNtrk3 and downstream targets including log2 fold-change (log2FC) expression. Shape legend: Circles, circRNA; rectangles, mRNA. Node colour gradients are proportional to log2FC. Edge length/colour gradients are proportional to correlation (shorter = greater correlation). (**C**) Cross-validation model for circRNA–mRNA target interactions. Component 1 (greatest factor of variation) and component 2 (second greatest factor of variation). Abbreviations: CircRNA, circular RNA; r, correlation; rCCA, Regularised Canonical Correlation Analysis; CV, cross-validation; HPH, Hypoxia-induced hypertension. Log2FC (pulmonary hypertension phenotype vs. control). Correlation generated by mixOmics (Version 6.1.1) using rCCA method; network generated using Cytoscape (Version 3.10.2).

Co-expression analysis was used to assess whether the prioritised H_PRN circRNA candidates showed expression patterns consistent with their predicted mRNA target networks. CircCnot6l showed moderate-to-strong positive correlations with several predicted downstream mRNAs, including *C1qtnf3* (r = 0.81), while circNtrk3 showed strong correlations with all five predicted mRNA targets (r > 0.85; Figure 4A,B). Regularized Canonical Correlation Analysis (rCCA) analysis further supported coordinated expression between circNtrk3 and its predicted target set, while circCnot6l showed greater dispersion along the second component (Figure 4C). These findings provide co-expression support for the prioritised candidate networks but do not establish direct regulatory interactions.

### 2.5. Exploratory Co-Expression Module Analysis of H_PRN Target Genes

To further contextualise the prioritised H_PRN mRNA targets, weighted gene correlation network analysis (WGCNA) was performed as an exploratory analysis of differentially expressed genes in the H_PRN dataset (Table S4). This analysis was used to assess whether predicted circCnot6l- and circNtrk3-associated mRNA targets were located within highly connected co-expression modules related to the hypoxia-induced pulmonary hypertension (HPH) phenotype.

The WGCNA analysis identified multiple co-expression modules, with module eigengenes showing clear hierarchical clustering (Figure S5A,B). The largest module was MEturquoise, which contained 1254 of 2762 genes included in the analysis. Most modules showed moderate-to-strong internal connectivity, although MEpink showed weaker connectivity compared with the other modules (Figure S5C). These results indicate that the H_PRN differentially expressed genes could be organised into distinct co-expression modules.

Module–trait correlation analysis showed that each module was significantly associated with the HPH phenotype (Figure S6, *p* < 0.01). MEblack, MEyellow and MEbrown showed the strongest module–trait associations (*p* < 0.001), suggesting that genes within these modules may be particularly relevant to the H_PRN hypoxia response. However, because the analysis was based on a binary phenotype comparison and a small sample size, these module–trait associations should be interpreted as exploratory rather than as independent evidence of regulatory function.

The predicted circCnot6l/miR-204-5p- and circNtrk3/miR-205-5p-associated mRNA targets generated from unpaired samples showed strong gene–trait correlations within the H_PRN dataset. circCnot6l-associated targets were positively correlated with the HPH phenotype, with all 19 targets showing HPH correlation coefficients greater than 0.8 (Figure S7B; Table 2). In contrast, circNtrk3-associated targets were negatively correlated with the HPH phenotype, with all five targets showing strong inverse correlations in Table 2. These results are consistent with the direction of differential expression observed for the two unmatched circRNA–miRNA and miRNA–mRNA predictive axes.

Most circCnot6l-associated target genes were assigned to MEturquoise, with 18 of 19 targets falling within this module (Table 2). This partly reflects the large size of MEturquoise, but also indicates that the circCnot6l-associated target set occupies a coherent region of the H_PRN co-expression network. In contrast, circNtrk3-associated targets were distributed across MEbrown, MEgreen and MEpink, with three of five targets—Cgnl1, Lsamp and Angpt2—assigned to MEbrown. Given the strong module–trait association observed for MEbrown, these genes may represent higher-priority downstream candidates for further investigation.

All predicted target genes listed in Table 2 showed high module membership values, suggesting that they were well connected within their assigned modules. Together, these results suggest that the predicted circCnot6l/miR-204-5p- and circNtrk3/miR-205-5p-associated mRNA targets generated from unpaired samples occupy highly connected regions of the H_PRN co-expression network. However, given the small sample size and binary phenotype contrast, these findings should be interpreted as exploratory support for candidate prioritisation rather than definitive evidence that these genes act as regulatory hub genes.

### 2.6. Protein Interaction and Pathway Enrichment Analysis of Prioritised H_PRN mRNA Targets

Protein–protein interaction analysis was performed to identify pathways represented among the prioritised H_PRN mRNA targets and their interacting proteins. This analysis was used as a downstream exploratory step to contextualise the unpaired and predicted circCnot6l/miR-204-5p- and circNtrk3/miR-205-associated mRNA targets within broader protein interaction networks.

**Figure 5:**
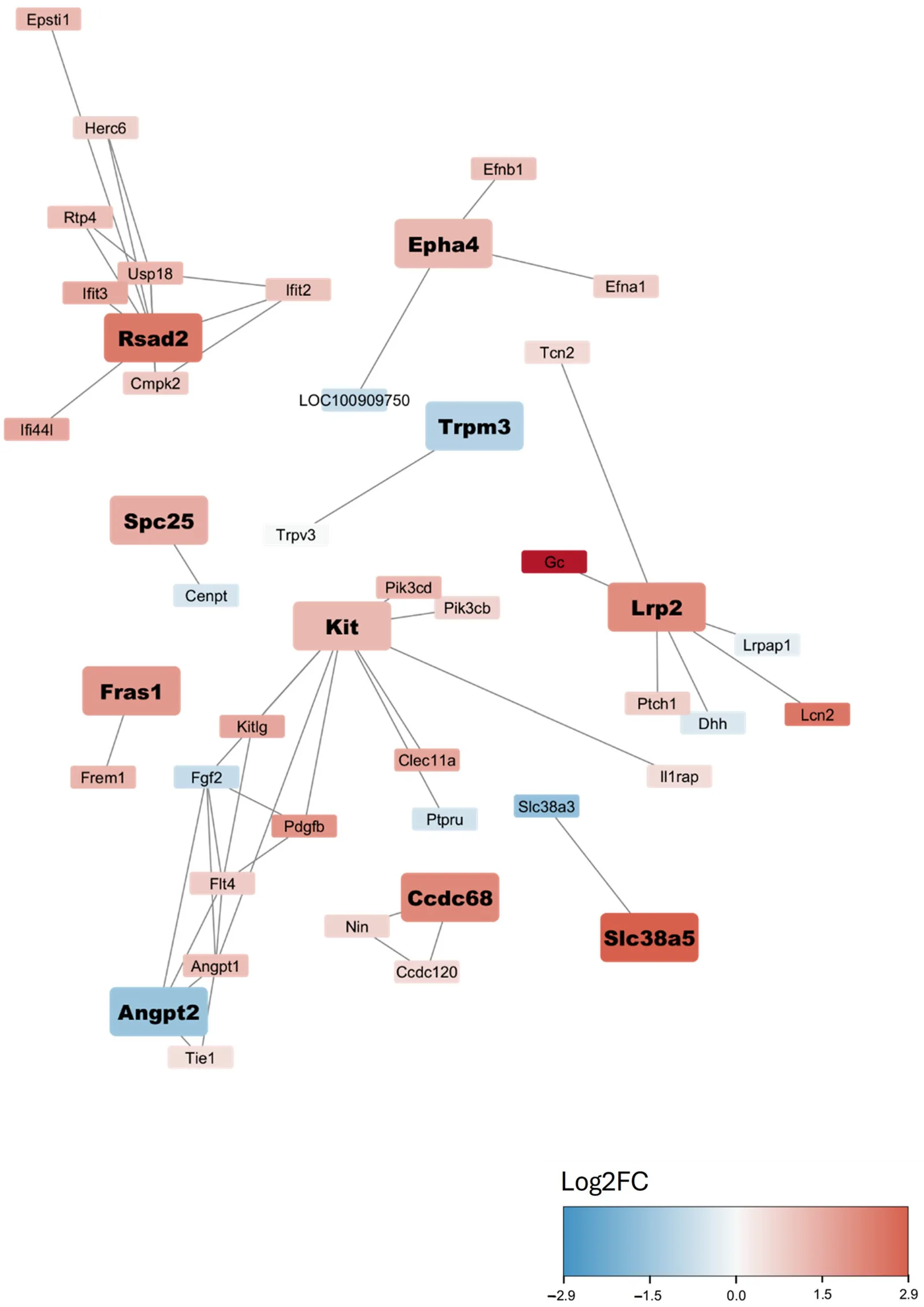
Protein–protein interaction network of prioritised H_PRN mRNA targets and interacting proteins**.** Protein–protein interaction analysis was performed using stringAPP. Enlarged and bolded nodes indicate prioritised messenger RNA (mRNA) targets from the predicted circCnot6l- and circNtrk3-associated networks. Transcript-level log2 fold-change (log_2_FC) values from PRJNA809145 (H_PRN) were mapped onto the corresponding protein nodes to visualise mRNA expression direction and magnitude; these values do not represent measured protein abundance. Interactions were filtered using a confidence cutoff of 0.8 and Benjamini-Hochberg adjusted *p*-value (FDR *q*) < 0.05. The network was visualised in Cytoscape (Version 3.10.2).

The resulting candidate network from unmatched samples highlighted interaction clusters involving Kit and Angpt2, with additional interaction support around Rsad2, Epha4 and Lrp2 (Figure 5). In contrast, Trpm3, Fras1, and Spc25 showed limited interaction support under the applied confidence threshold. Mapped transcript-level log_2_FC values suggested potential upregulation of several network-associated genes in HPH relative to control tissue. However, these values were derived from transcriptomic data and should not be interpreted as direct measurements of protein abundance.

Functional enrichment analysis identified several signalling pathways associated with the prioritised H_PRN candidate target genes and their interacting proteins. Downregulation of Angpt2 and Fgf2, together with upregulation of Efna1, Flt4, Pdgfb, Angpt1, Pik3cb, Kitlg, Pik3cb and Tcn2, was associated with enrichment of Rap1, Ras, and PI3K-Akt signalling pathways (Table 3). A similar set of genes, including Angpt2, Fgf2, Efna1, Fit4, Pdgfb, Angpt1, Kitlg, il1rap and Tcn2, was associated with MAPK signalling. Finally, upregulation of Tnc2 and Kitlg, together with downregulation of Ptpru, was associated with enrichment of SCF-KIT signalling.

Together, these findings suggest that the prioritised H_PRN mRNA predicted targets from unmatched samples are linked to protein interaction networks and pathways involved in cell adhesion, proliferation, migration, survival and angiogenesis. These pathway-level results provide additional biological context for the predicted circCnot6l- and circNtrk3-associated networks.

## 3. Discussion

CircRNAs are increasingly recognised as regulators of PH-associated vascular remodelling through their modulation of miRNA–mRNA networks that influence cell proliferation, migration, and apoptosis. For example, hsa_circ_0016070 promotes pulmonary artery smooth muscle cell (PASMC) proliferation by upregulating CCND1 via the miR-942/CCND1 axis [22]. Similarly, circST6GAL1 has been reported to enhance cell proliferation and migration while suppressing apoptosis through regulation of the miR-509-5p/MCTP2 pathway [23]. In contrast, circALMS1 inhibits PASMC proliferation and migration through the miR-17-3p/YTHDF2 axis [24], while hsa_circ_0001402 exerts comparable anti-remodelling effects during pulmonary hypertensive neointimal hyperplasia [25]. More recently, circNFXL1 was shown to attenuate right ventricular hypertrophy and pulmonary vascular remodelling in a mouse model of PH following intranasal liposomal delivery [26].

Consistent with this growing body of evidence, the present study performed an exploratory circRNA analysis across three independent datasets. The most robust and biologically interpretable signal was identified in the H_PRN hypoxia-exposed pulmonary artery dataset, leading to the prioritisation of circCnot6l and circNtrk3 for further investigation. Given that pulmonary artery tissue is the primary site of vascular remodelling in PH, it may provide greater biological relevance than whole-lung tissue, thereby contributing to the stronger signal observed. Furthermore, differences in disease models (hypoxia versus monocrotaline), sequencing depth, sample size, and circRNA detection sensitivity may account for the limited overlap in candidate circRNAs across datasets.

A total of 12,713 circRNAs were identified in the H_PRN dataset, of which circCnot6l, circNtrk3, and circSmoc1 were significantly differentially expressed. None of these circRNAs were differentially expressed in the lung tissue datasets (M_PRN and H_GSE) under the current filtering criteria, which may reflect the high cellular heterogeneity of the total lung tissue (>40 cell types) compared with TPAT [27,28,29,30].

Functional enrichment analysis indicated increased angiogenesis, vascular regulation, cell adhesion, and cell migration, consistent with activation of hypoxia-inducible factors (HIFs) during hypoxia and the development of vascular remodelling [31,32,33,34]. Downregulation of supramolecular fibre organisation may further suggest loss of vascular integrity through altered collagen, actin, and elastin organisation [35,36,37].

CircCnot6l and circNtrk3 were predicted to interact with miR-204-5p and miR-205, respectively. In contrast, the predicted circSmoc1 target miR-329-3p showed only marginal significance, likely due to the small sample size [38]. Although circCnot6l possessed a larger number of predicted targets, circNtrk3 demonstrated stronger overall associations with downstream gene expression.

WGCNA was used to provide additional support for circRNA target prioritisation. Consistent with previous studies, most genes clustered within the MEturquoise module [39,40,41]. MEbrown exhibited the strongest module–trait relationships, supporting connectivity among *Cgnl1*, *Lsamp*, and *Angpt2*. However, universal module significance may reflect limited sensitivity resulting from the small sample size (*n* = 7) and the use of binary trait data [42,43,44,45]. Notably, all predicted circNtrk3 targets and most circCnot6l targets ranked within the upper range of gene–phenotype correlations.

Reduced miR-204-5p expression was consistent with findings in kidney disease, tumour progression, and arthritis [46,47,48,49,50,51,52]. Conversely, increased miR-205 expression has been associated with inhibition of cell proliferation, migration, invasion, apoptosis, and epithelial–mesenchymal transition, although its role in differentiation appears to be context-dependent [53,54,55]. Collectively, these findings could suggest that both miRNAs may contribute to pulmonary vascular remodelling.

Among circCnot6l targets, *C1qtnf3* was upregulated. Previous studies suggest that *C1qtnf3* may contribute to cardiac hypertrophy and dysfunction [56], but may also exert anti-inflammatory, anti-fibrotic, and vasculoprotective effects through FGFR1-Ras/PI3K/Akt signalling [57,58,59,60]. Thus, its role in TPAT remodelling remains unclear.

Among circNtrk3 targets, *Cgnl1* was potentially downregulated and ranked among the strongest HPH-associated genes. Reduced *Cgnl1* expression has been linked to impaired endothelial junction stability, reduced angiogenesis, and disrupted cell–matrix adhesion [61,62,63,64,65,66,67], potentially contributing to impaired vessel formation and increased vascular resistance [68,69,70,71,72]. Similarly, *Angpt2* was downregulated and clustered with *Cgnl1* within MEbrown. Given the established roles of *Angpt2* in angiogenesis, vascular remodelling, inflammation, and CVD [73,74], combined inhibition of *Cgnl1* and *Angpt2* may indicate impaired vascularisation despite hypoxia-induced VEGF signalling. This interpretation is supported by the upregulation of pro-angiogenic genes such as *Slc38a5* and *Clec14a* [75,76].

Finally, protein–protein interaction and pathway analyses identified Angpt2 as a central network component linked to Rap1, Ras/MAPK, PI3K-Akt, and ERK signalling pathways. These pathways regulate cell proliferation, survival, migration, differentiation, metabolism, and angiogenesis [77,78,79,80,81]. Together, these findings could suggest that circCnot6l and circNtrk3 may contribute to pulmonary vascular remodelling through regulation of miR-204-5p- and miR-205-mediated gene networks and downstream signalling pathways. The observed inhibition of Ptpru, previously implicated in elevated right ventricular pressure and vascular wall thickening in HPH, further supports this model [82].

This study has several limitations. First, although the data underwent stringent filtering and multi-tool quantification, in vivo validation was not performed; therefore, the results should be interpreted as a computational prediction only. Second, different miRNA-seq and RNA-seq samples were used during the original study (PRJNA809145), introducing potential bias. Third, continuous variables, such as telemetric blood pressure or vascular wall thickness, would have provided greater statistical certainty during eigengene modelling (WGCNA). Fourth, the complexity of competing endogenous RNA (ceRNA) networks, particularly the contribution of long non-coding RNAs (lncRNAs), was not explored and may have influenced target expression. Fifth, protein expression was inferred indirectly from mRNA abundance and does not account for post-transcriptional regulation or protein-level modifications. To validate both expression and function, transcriptome-wide analysis of ceRNA networks should be performed alongside in vitro validation studies. Finally, the findings are primarily based on the H_PRN dataset, are computational in nature, rely on relatively small sample sizes, and require experimental validation of circRNA expression, back-splice junctions, miRNA interactions, and downstream functional effects.

## 4. Materials and Methods

### 4.1. Data Collection

To investigate the role of circular RNA in PH, a comprehensive search of publicly available datasets (NCBI) was conducted using the following searching terms:

(PULMONARY[All Fields] AND HYPERTENSION[All Fields] AND (“Rattus”[Organism] OR RATTUS[All Fields])) AND (“bioproject sra”[Filter] AND “org mammals”[Filter]).

CircRNAs are considered a subclass of lncRNA; therefore, pair-end bulk RNA-seq data with a depth of 25M+ and an RNA integrity number (RIN) of >8 were collected, in accordance with CCR Collaborative Bioinformatics Resource (CCBR) for lncRNA analysis [83]. Further, Ribo-Zero depletion must be done to enrich circRNA detection and reduce ribosomal RNA (rRNA) noise [84,85,86]. In total, three datasets were revealed [19,20,21] (Table 4 and Table S5–S7).

### 4.2. In Silico Quantification

Rat genome assembly 6.0 (Rnor_6.0) was retrieved from NCBI, then RNA-seq fastq files were validated with full-quality base scores as part of the Nextflow pipeline (multiqc v1.27). After, quantification of circRNA back-splice junctions was done using CIRI2 Full (Version 2.1.1) and Nextflow (Version 24.10.2) container-based nfcore/circrna (dev) pipelines (CIRIquant v1.1.3, circRNA_finder v1.2 and find_circ v1.2) [17]. Next, manual adapter trimming was done for raw miRNA-seq data (*n* = 5 control and *n* = 5 (HPH)), and quantification was done using Nextflow nfcore/srmaseq (Version 2.3.1) [87]. Lastly, mRNA-level expression was calculated during circRNA quantification (nfcore/circrna (dev)) by psirc-quant.

CIRI2, CIRIquant, circRNA_finder and find_circ share the primary function of identifying and quantifying back-splice junction (BSJ) reads; however, they differ in their alignment strategies and computational algorithms (Table S3). Accordingly, circRNAs quantified by more than one tool demonstrate greater statistical confidence.

In the literature, CIRI2 has demonstrated strong benchmark performance [88,89] and has been utilised in several key studies investigating circRNA expression in renal disease and hypertension [90,91]. Early and recent publications using CIRI2 have collectively received more than 1000 citations [88,89], highlighting its widespread adoption within the scientific community. In contrast, CIRIquant offers improved accuracy in BSJ read detection and reduction of false-positive identification, largely due to its split-mapping functionality. This approach enables detected BSJ reads to be realigned against the reference genome and pseudo-circular transcripts, facilitating the correction of computational errors through an advanced algorithmic framework [15].

circRNA_finder is an established tool for circRNA identification and quantification. Although its pipeline is less complex than CIRI2 and CIRIquant, circRNA_finder utilises spliced transcript alignment to a reference genome through STAR for indexing and alignment. According to a cross-comparison study conducted by Schaarschmidt et al. [92], STAR mapped 99.5% of the genome compared with 95.7% using BWA. While both approaches demonstrate marginal deviation, robust alignment remains essential for reducing FDR and improving output quality. Moreover, analyses of HEK293 cells have demonstrated high efficiency in filtering linear transcripts, duplicate reads, and other sources of false-positive detection [93].

Finally, find_circ was the first publicly available pipeline designed for circRNA identification and has been cited nearly 8000 times [94,95]. It employs a de novo algorithm and Bowtie2 to quantify de novo circular RNAs from RefSeq datasets. Although it is now considered less advanced than more recent software that incorporates STAR alignment, Bowtie2 provides a statistical advantage during cross-tool comparisons and candidate selection by introducing a fourth alignment mode. The utility of this approach was demonstrated in a 2018 study in which the authors used CIRCexplorer2, circRNA_finder, CIRI, find_circ, and MapSplice2 to identify circNFIX, a biomarker associated with glioma progression through modulation of the miR-34a-5p/NOTCH1 axis [96].

### 4.3. Differential Expression Analysis

Differential expression analysis was conducted using edgeR (Version 4.0.16) with quasi-likelihood dispersion estimation and trimmed mean of M values (TMM) normalisation. Here, dispersion parameters for each circRNA/miRNA and gene are estimated with the Cox–Reid common dispersion [97] and applied to a negative binomial generalised linear model. This ensures that differences in expression that are consistent between replicates are more highly weighted to ensure differential expression is not guided by outliers. *p*-values were adjusted for multiple testing using the Benjamini–Hochberg correction . Count data for each tool was then filtered by GLMM (glmmTMB ver 1.1.11) using *Count ~ Group + (1|Tool)* with a negative binomial distribution and zero inflation. Finally, graphics were generated using ggplot2 (Version 3.5.1) in R (Version 4.3.2).

### 4.4. Candidate Selection Criteria

To be considered a hit, circRNA junctions required a minimum of 2 reads; otherwise, the value was set to “0”. Moreover, each circRNA was required to show at least 5 counts per million (CPM) in ≥2 control/pulmonary hypertensive samples across ≥2 identification/quantification tools. Finally, glmmTMB was used to ensure circRNA with a low read count and/or poor inter-tool concordance were filtered out Pr(>|z|) ≥ 0.05.

Differentially expressed circRNA, miRNA and mRNA must have an FDR *q*-value < 0.05 (1 + tool), and a log2FC > 1 (log2FC > 0.95) may be considered. If the FDR *q*-value > 0.05 and *p*-value < 0.05, transcripts are considered nominally differentially expressed [98].

To ensure clinical relevancy, differentially expressed candidates must be translatable to humans; this is determined by CircAtlas 3.0, which evaluates sequence and expression conservation by generating a multiple conservation Score (MCS).

### 4.5. CircRNA/miRNA/mRNA Target Prediction

To identify circRNA–miRNA interactions, integrated CircAtlas 3.0 webtools—PITA, miRanda (Version 3.3a) and Targetscan (Version 7.0)—were used. CircRNAs with at least 1 MRE binding region detected by ≥2/3 predictive softwares were considered a downstream target. To identify miRNA–miRNA interaction, miRTargetLink2.0 (Version 79.0.3945.88) was used to scan for mRNA targets validated by experiment (miRTarBase (Version 8.0)) or predicted by miRDIP (Version 4.1) and miRDB software (Version 6.0).

### 4.6. Gene and Protein Ontology

Gene ontology was done to reveal enriched single-ranked processes using Gene Ontology enRIchment anaLysis and the visuaLizAtion tool (GOrilla, 8 March 2013 release). Subsequently, protein ontology was done using stringApp (Version 2.1.1), with a confidence threshold cutoff of 0.8/1 to ensure pathways maintain high certainty.

### 4.7. Multivariate/Covariate Analysis

Given samples used for miRNA-seq analysis differ from those used during circRNA and mRNA analysis (study PRJNA809145, *n*(miRNA-seq) = 10 (control, NC-5–NC-9; experimental, HPH-10–HPH-14), *n*(RNA-seq) = 7 (control, NC-1–NC-4; experimental, HPH-17–HPH-19)), a covariate approach was taken to analyse the relationship between circRNA- and mRNA-level expression to reduce potential bias. Initially, circRNA and mRNA count data underwent normalisation by variance stabilisation transformation (VST) using DESeq2 (Version 1.42.1). Then, both the count and sample phenotype were piped into MixOmics using a standard correlation cutoff of ≥ 0.5 for components 1 and 2. Covariate analysis (circRNA–mRNA axis) was conducted using a Regularised Canonical Correlation Analysis model (rCCA) with a ridge penalty (MixOmics (Version 6.1.1)) to handle high-dimensional data [99]. Moreover, the threshold cutoff was set (0.5/1) to ensure stringent correlation.

To investigate the gene–trait association with the phenotype of PH, module eigengenes were generated with WGCNA (Version 1.72-1) using binary trait data. Here, regression scatterplots were used to determine how well genes cluster together within modules. This is calculated by plotting intramodular connectivity (kWithin; x-axis) against gene significance (SIG; y-axis). After, graphics for multivariate analysis, protein ontology and target prediction were generated using Cytoscape (Version 3.10.2).

## 5. Conclusions

No differentially expressed circRNAs were identified in the lung tissue datasets (M_PRN and H_GSE). However, analysis of TPAT from the H_PRN dataset revealed significant differential expression of the circCnot6l/miR-204-5p and circNtrk3/miR-205 regulatory axes. Subsequent candidate network and unmatched pathway analyses identified several downstream mRNA targets previously implicated in PH pathogenesis, including *Cgnl1*, *Angpt2 *and* C1qtnf3*, highlighting their potential relevance to the hypoxia-induced PH phenotype. Furthermore, gene ontology and protein–protein interaction analyses revealed enrichment of biological processes and signalling pathways associated with vascular remodelling.

Taken together, this study is the first to identify circCnot6l and circNtrk3 as candidate regulatory molecules associated with key pathological processes in hypoxia-induced PH, including angiogenesis, cell proliferation, adhesion, migration and vascular remodelling. By leveraging the novel container-based Nextflow nf-core/circrna pipeline incorporating CIRI2, this work provides a robust framework for circRNA discovery in PH. Future studies should focus on experimental validation of the circCnot6l/miR-204-5p and circNtrk3/miR-205 regulatory axes in vitro and in vivo, as well as assessing their conservation and clinical relevance in human PH.

## Figures and Tables

**Table 1:** Prioritised circRNA candidates identified across rat PH datasets, with robust candidates detected primarily in H_PRN pulmonary artery tissue.

CircAtlas 3.0 ID	Tools/4	Dataset	Average log2FC	Average *p*-Value	Lowest Tool FDR	glmmTMB Pr(>|z|)
*circSmoc1_0009*	4	H_PRN	−1.406	1.35 × 10^−3^	2.71 × 10^−7^	1.46 × 10^-16^
*circNtrk3_0005*	4	H_PRN	−1.727	6.69 × 10^−5^	2.61 × 10^−4^	2.86 × 10^-32^
*circCnot6l_0006*	3	H_PRN	1.355	9.02 × 10^−3^	0.0151	2.36 × 10^−6^
*circMap3k5_0005*	2	H_PRN	1.116	7.58 × 10^−3^	0.269	3.72 × 10^−3^
*circSmoc1_0002*	2	H_PRN	−1.417	0.0140	0.269	1.02 × 10^−8^
*circINT_000237*	4	H_PRN	−1.490	0.0227	0.345	9.69 × 10^−7^
*circSlc16a10_0003*	4	H_PRN	2.374	0.0188	0.421	3.76 × 10^−10^
*circZfp148_0001*	2	H_PRN	−1.606	0.0271	0.551	2.23 × 10^−3^
*circNsd1_0007*	2	H_PRN	−1.235	0.0348	0.551	4.18 × 10^−5^
*circMagi1_0007*	2	H_PRN	−1.694	0.0396	0.551	4.22 × 10^−6^

CIRIquant, circRNA_finder, find_circ; 4/4 indicates complete detection. Dataset, study dataset (Materials and Methods: Data Collection). Average log2FC, sum of Log2 fold-change (log2FC) expression/number of detected tools. Average *p*-value, sum of *p*-value/number of detected tools. Lowest tool FDR, lowest singular false discovery rate (FDR) value across each tool. GlmmTMB, Generalized Linear Mixed Models using Template Model Builder. Pr(>|z|) < 0.05, significant alignment between tools. CircRNA, circular RNA; DE circRNA, differentially expressed circRNA (Bold; *n* = 3, *q* < 0.05, log2FC > 1, Tool 1+); NDE circRNA, nominally differentially expressed circRNA (*n* = 7, *p <* 0.05, log2FC > 1, Tool 1+); H_PRN, study PRJNA809145; M_PRN, study PRJNA732522. Log2FC (pulmonary hypertension phenotype vs. control).

**Table 2:** Target expression and connectivity.

Parent circRNA	Differential Expression			WGCNA			
	HPH vs. Control			Module Connectivity		Gene–Trait	
	Gene	Log2FC	*q*-Value	Module	MM	HPH(r)	*p*-Value
CircNtrk3	*Cgnl1*	−1.316	0.0004	brown	0.982	−0.993	7.090 × 10^−6^
	*Lsamp*	−2.269	0.0016	brown	0.968	−0.964	4.695 × 10^−4^
	*Trpm3*	−1.085	0.0027	green	0.968	−0.947	1.204 × 10^−3^
	*Angpt2*	−1.502	0.0022	brown	0.975	−0.941	1.570 × 10^−3^
	*Atp2b2*	−1.530	0.0077	pink	0.953	−0.909	4.613 × 10^−3^
CircCnot6l	*Arap2*	1.930	0.0006	turquoise	0.981	0.978	1.393 × 10^−4^
	*Tmem71*	2.178	0.0026	turquoise	0.952	0.966	3.990 × 10^−4^
	*Epha4*	1.226	0.0013	turquoise	0.986	0.955	7.963 × 10^−4^
	*Slc38a5*	2.921	0.0022	turquoise	0.998	0.939	1.676 × 10^−3^
	*Plch1*	1.597	0.0055	turquoise	0.936	0.930	2.365 × 10^−3^
	*Ripk4*	1.638	0.0049	turquoise	0.990	0.928	2.590 × 10^−3^
	*Fras1*	1.815	0.0039	turquoise	0.979	0.911	4.316 × 10^−3^
	*Ccdc68*	2.140	0.0069	turquoise	0.978	0.909	4.610 × 10^−3^
	*Tox3*	1.964	0.0033	turquoise	0.980	0.899	5.824 × 10^−3^
	*Rsad2*	2.440	0.0028	turquoise	0.944	0.896	6.362 × 10^−3^
	*Elavl2*	2.822	0.0090	turquoise	0.880	0.891	7.140 × 10^−3^
	*Gpsm3*	1.056	0.0090	turquoise	0.959	0.882	8.600 × 10^−3^
	*Rtkn2*	2.320	0.0087	turquoise	0.965	0.879	9.092 × 10^−3^
	*Rgs12*	1.105	0.0086	turquoise	0.962	0.870	1.097 × 10^−2^
	*Lrp2*	2.046	0.0129	turquoise	0.959	0.861	1.285 × 10^−2^
	*C1qtnf3*	2.176	0.0072	blue	0.930	0.856	1.393 × 10^−2^
	*Kit*	1.216	0.0142	turquoise	0.962	0.826	2.204 × 10^−2^
	*Spc25*	1.425	0.0389	turquoise	0.899	0.814	2.588 × 10^−2^
	*Clec14a*	1.118	0.0216	turquoise	0.945	0.803	2.981 × 10^−2^

This table represents a summary of target gene expression, module allocation, membership and gene–trait association. “Module” represents parent module generated by Weighted Gene Co-expression Network Analysis (WGCNA) (Figure S6) (“turquoise”, “blue”, “brown”, “green” or “pink”). Module membership (MM) = how well gene target connects within parent module 0–1. HPH(r) = gene–trait correlation for hypoxia-induced hypertension (HPH, 1 = compete correlation with phenotype). *p*-value = Pearsons test for gene–trait significance (*p* < 0.05 = significant). Rank is based on HPH(r) value (1 = greatest correlation, −1 = greatest inverse correlation, 0 = no correlation). Log2FC (HPH vs. control). “*q*-value” = FDR adjusted *p*-value (*p* < 0.05 = significant). Log2FC = Log2 fold-change; FDR, false discovery rate; HPH, hypoxia-induced hypertension. WGCNA analysis conducted using R. (Version 4.3.2).

**Table 3:** Enriched pathways associated with prioritised H_PRN mRNA targets and their interacting proteins.

Candidate Protein/Cluster	Pathway	Function	*p*-Value
Angpt2 + 9	Rap1 signalling pathway	Cell adhesion, proliferation and migration	6.46 × 10^−9^
Angpt2 + 9	Ras signalling pathway	Cell growth, proliferation, differentiation and survival	1.69 × 10^−8^
Angpt2 + 9	PI3K-Akt signalling pathway	Cell growth, survival, proliferation and metabolism	7.42 × 10^−7^
Angpt2 + 8	MAPK signalling pathway	Stress response, cell proliferation, differentiation and apoptosis	4.18 × 10^−6^
Tcn2 + 2	Signalling by SCF-KIT	Cell proliferation, migration and survival	8.57 × 10^−3^

Pathways were identified from the protein–protein interaction network and ranked by enrichment *p*-value. The listed functions summarise major biological processes represented by each pathway. Transcript-level log_2_FC values from H_PRN were mapped onto the corresponding protein nodes to illustrate the direction and magnitude of transcriptomic changes. These values were derived from mRNA expression data and should not be interpreted as direct measurements of protein abundance. PPI, protein–protein interaction; MAPK, mitogen-activated protein kinase; PI3K-Akt, phosphoinositide 3-kinase/protein kinase B; SCF-KIT, stem cell factor–KIT signalling.

**Table 4:** Study metadata collected from NCBI on pulmonary hypertension in rats.

Study	Rat Type	Age	Control	Experiment	Tissue/RNA	DE Definition	Link
PRJNA809145/ H_PRN	Male Sprague-Dawley rats	8 weeks exposure begins, 12-week harvest	Normoxia 21% O_2_ (*n* = 4)	Hypoxia 10% O_2_ (*n* = 3)	Total RNA from pulmonary arteries	CircRNA/ LncRNA— *p <* 0.05, log2FC > 1	Link1
GSE159668/ H_GSE	Male Sprague-Dawley rats	6–8 weeks exposure begins, 9–11-week harvest	Normoxia 21% O_2_ (*n* = 3)	Hypoxia 9.5–10.5% O_2_ (*n* = 3)	Total RNA from right lung tissues	LncRNA— *p <* 0.05, log2FC >1	Link2
PRJNA732522/ M_PRN	Male Wistar Rats	4 weeks inoculation, 12-week harvest	Healthy control (saline, *n* = 3)	Single dose—Monocrotoline @ 60mg/kg (*n* = 3)	Total RNA from lung tissue	LncRNA— *p <* 0.05, log2FC > 2	Link3

Each study follows RNA-Seq data collection guidelines (CCBR) for long non-coding RNA (lncRNA) analysis and uses ribo zero for ribosomal RNA (rRNA) depletion. Abbreviations: circRNA, circular RNA; log2FC, Log2 fold-change; DE, differentially expressed; mg, milligrams; kg, kilograms. *p < 0.05* is significant. Link1: https://www.ncbi.nlm.nih.gov/bioproject/PRJNA809145/(accessed on the 14 June 2025) from [19]; Link2, https://www.ncbi.nlm.nih.gov/geo/query/acc.cgi?acc=GSE159668 (accessed on the 25 June 2025) from [20]; Link3, https://www.ncbi.nlm.nih.gov/bioproject/PRJNA732522/ (accessed on the 17 June 2025) from [21].

## Data Availability

All data included in this study are publicly available on the following three websites: https://www.ncbi.nlm.nih.gov/bioproject/PRJNA809145/, 14 June 2025; https://www.ncbi.nlm.nih.gov/geo/query/acc.cgi?acc=GSE159668, 25 June 2025; https://www.ncbi.nlm.nih.gov/bioproject/PRJNA732522/, 17 June 2025.

## Supplementary Materials

The following supporting information can be downloaded at: https://www.mdpi.com/article/10.3390/ijms27188195/s1.

## Abbreviations

The following abbreviations are used in this manuscript:

CCBR	Centre for cancer research collaborative bioinformatics resource
CCND1	Cyclin D1
ceRNA	Competitive endogenous RNA
CircCnot6l_0006	CircCnot6l
CircNtrk3_0005	CircNtrk3
CircRNA	Circular RNA
CircSmoc1_0009	CircSmoc1
CPM	Counts per million
CVD	Cardiovascular disease
DE	Differentially expressed
DTC	Dynamic tree cut
FDR q	False Discovery Rate-adjusted p-value
GLMM	Generalised linear mixed model
GO	Gene Ontology
H_GSE	GSE159668
H_PRN	PRJNA809145
HPH	Hypoxia-induced pulmonary hypertension
Log2FC	Log2 fold-change
M_PRN	PRJNA732522
MCS	Multiple conservation score
MCTP2	Multiple C2 And Transmembrane Domain Containing 2
MD	Merged Dynamic
ME	Module eigengene
miRNA	Micro RNA
MM	Module membership
mRNA	Messenger RNA
NDE	Nominally differentially expressed
PAEC	Pulmonary artery endothelial cell
PASMC	Pulmonary artery smooth muscle cell
PCC	Pearson correlation coefficient (*p*)
PH	Pulmonary hypertension
Pr	Probability
rCCA	Regularised canonical correlation analysis
RIN	RNA integrity number
rRNA	Ribosomal RNA
SIG	Significance
TPAT	Total pulmonary artery tissue
TMM	Trimmed mean of M values
VST	Variance stabilisation transformation
WGCNA	Weighted Gene Co-Expression Network Analysis
