# Supplementary material for: Integrated Analysis of Rat Pulmonary Hypertension Datasets Identifies Candidate circRNA Networks in Hypoxia-Exposed Pulmonary Artery Tissue
Source: Int J Mol Sci. 2026 Sep 15;27(18):8195. doi: 10.3390/ijms27188195 (PMC13607602; doi:10.3390/ijms27188195)
Supplement: Supplementary file 1 [file ijms-27-08195-s001.zip › ijms-4474253-supplementary.pdf]

Supplementary Material

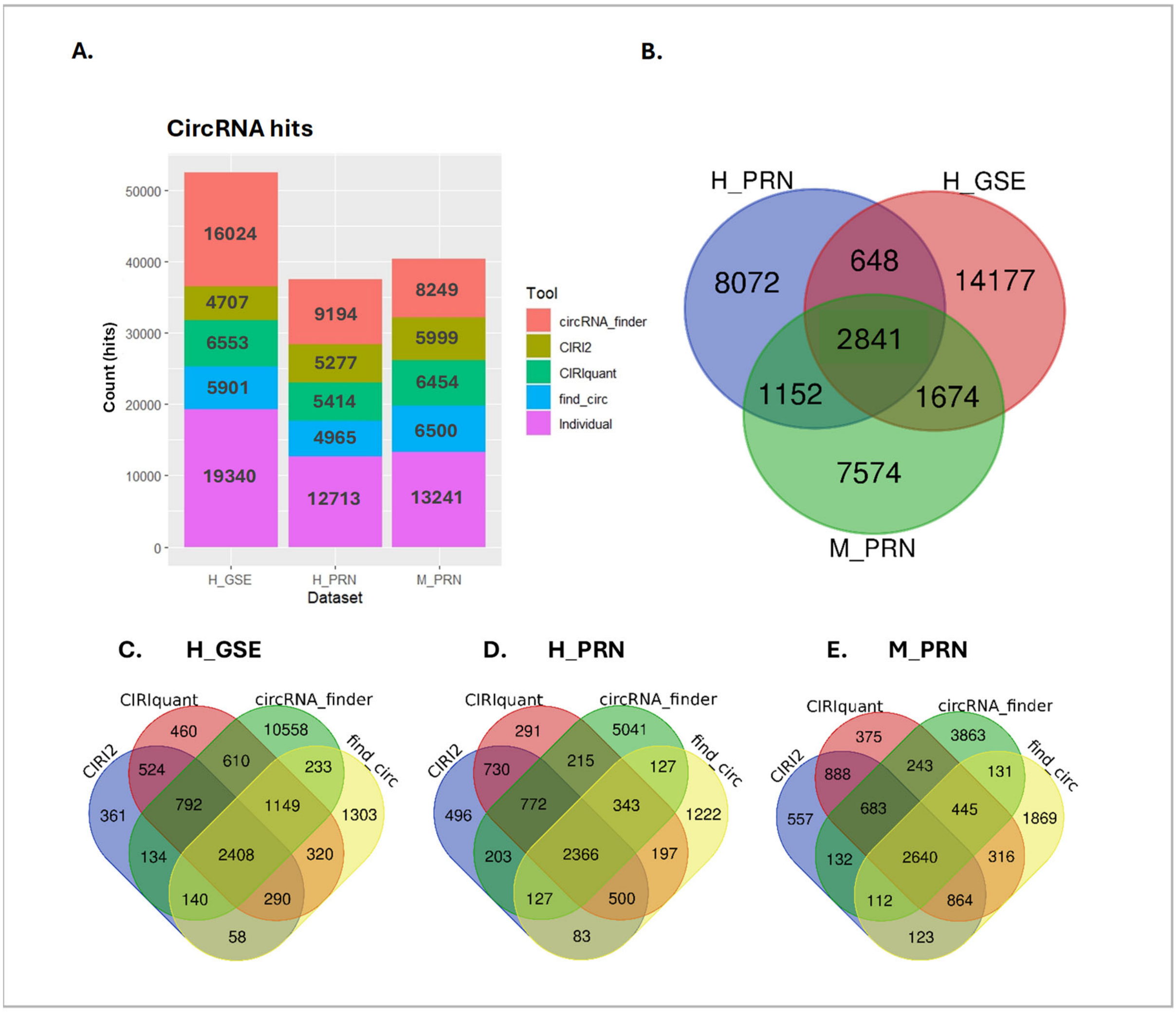

Figure S1. Compressive detection of circRNA across tool and dataset. Tool = ciriQuant, CIRI2, circRNA_finder and find_circ. Dataset = H_PRN = PRJNA809145, H_GSE = GSE159668 & M_PRN = PRJNA732522 (Methods Table 1). BSJ read(min) = 2. **A**) bar chart of circRNA hit discovery, "Individual" = total unique circRNA detected by one or more tools. **B**) Venn diagram: relation of circRNA hit between dataset. **C-E**) Venn diagram(s), relation of circRNA hit between tools and across datasets. Bar plot was generated using ggplot2 ver. 3.5.1 and R-studio. Venn diagrams were created using webtool: https://bioinformatics.psb.ugent.be/webtools/Venn/.

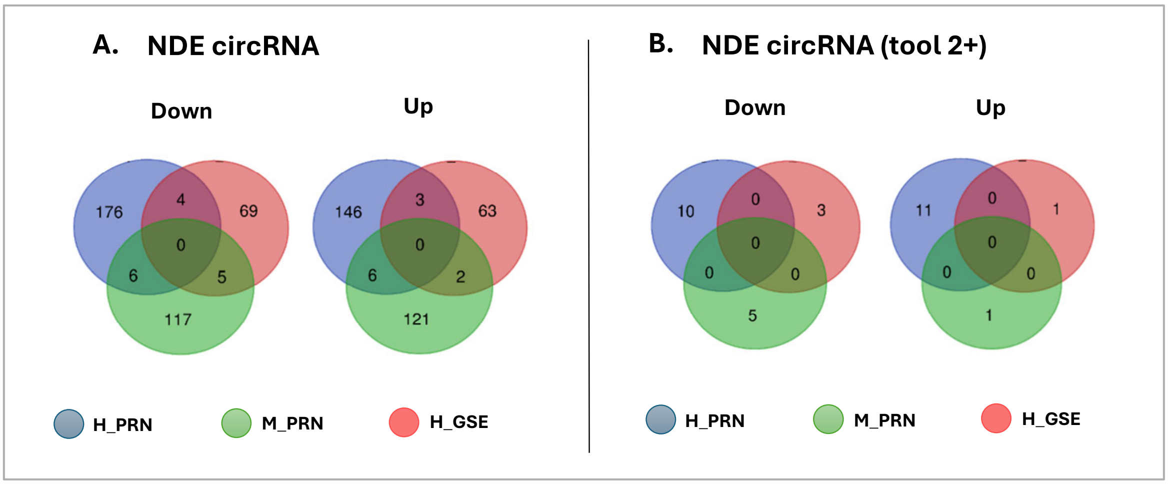

Figure S2. Evaluating hit crossover for nominally significant circRNA (p < 0.05). Tools (n = 4), CIRI2, circRNA_finder, CIRIquant, find_circ. **A**) Overlap of circRNA showing at least nominal differential expression (n(down) = 377, n(up) = 341, p < 0.05, log2FC > 1) across all three datasets (PRJNA809145 (H_PRN), GSE159668 (H_GSE), PRJNA732522 (M_PRN) (Methods Table 1). **B**) Overlap of circRNAs nominally differentially expressed (n(down) = 18, n(up) = 13, p < 0.05, log2FC > 1) detected by at least 2 quantification pipelines across all three datasets. MDS plots generated using limma ver. 3.53.2, Venn diagrams generated using webtool: https://bioinformatics.psb.ugent.be/webtools/Venn/.

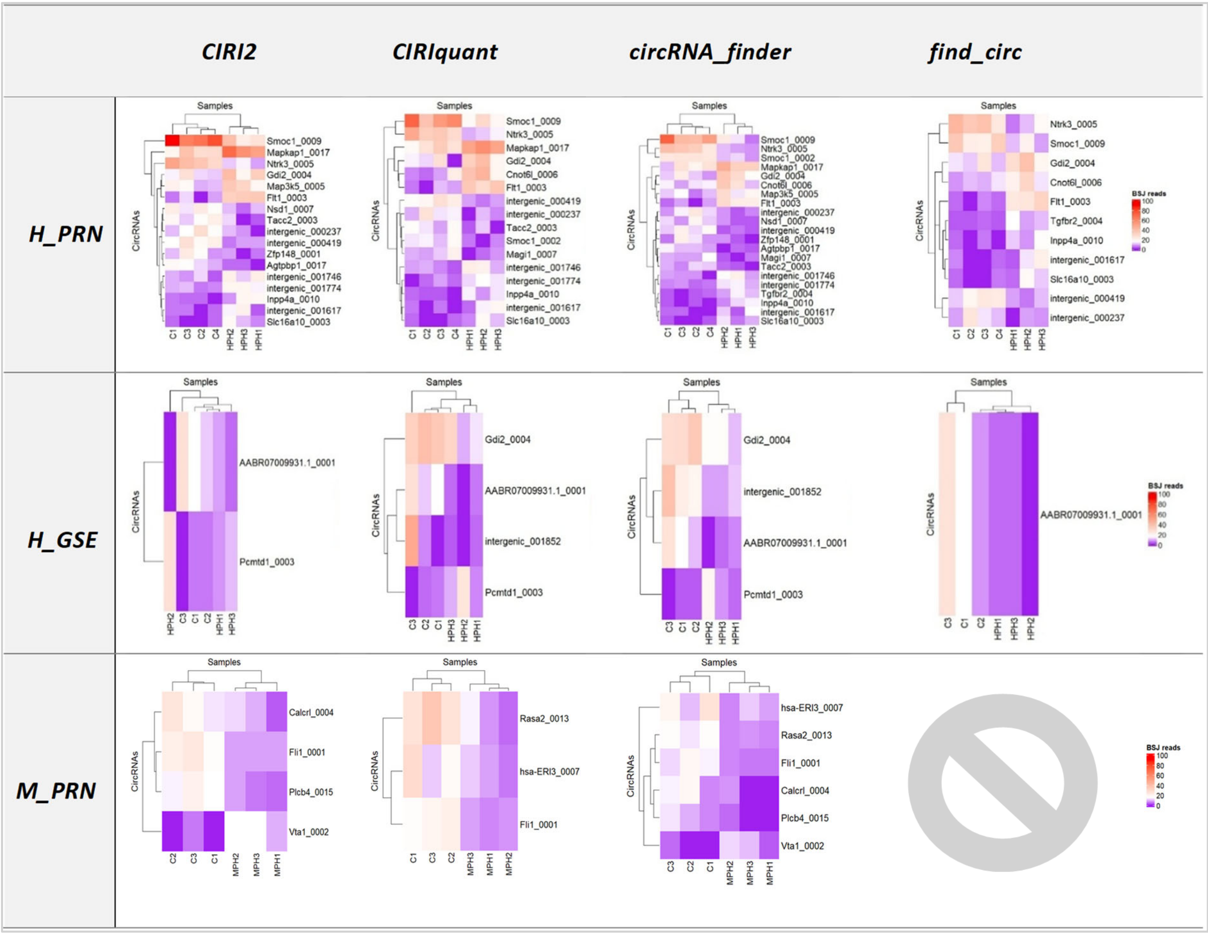

Figure S3. Heatmap series of nominally differentially expressed circRNA ($p < 0.05$, tool > 1) across tool and dataset. Each row represents a different dataset (H_PRN = PRJNA809145, H_GSE = GSE159668 & M_PRN = PRJNA732522). Each column represents a different tool used for circRNA quantification. Expression was generated using raw back splice junction read output provided by each tool. BSJ, back splice junction; C, control sample; HPH, hypoxia-induced pulmonary hypertension; MPH, monocrotaline-induced pulmonary hypertension. Heatmaps were generated by R-studio ver. 2025.05.0. and circlize ver. 0.4.16 package.

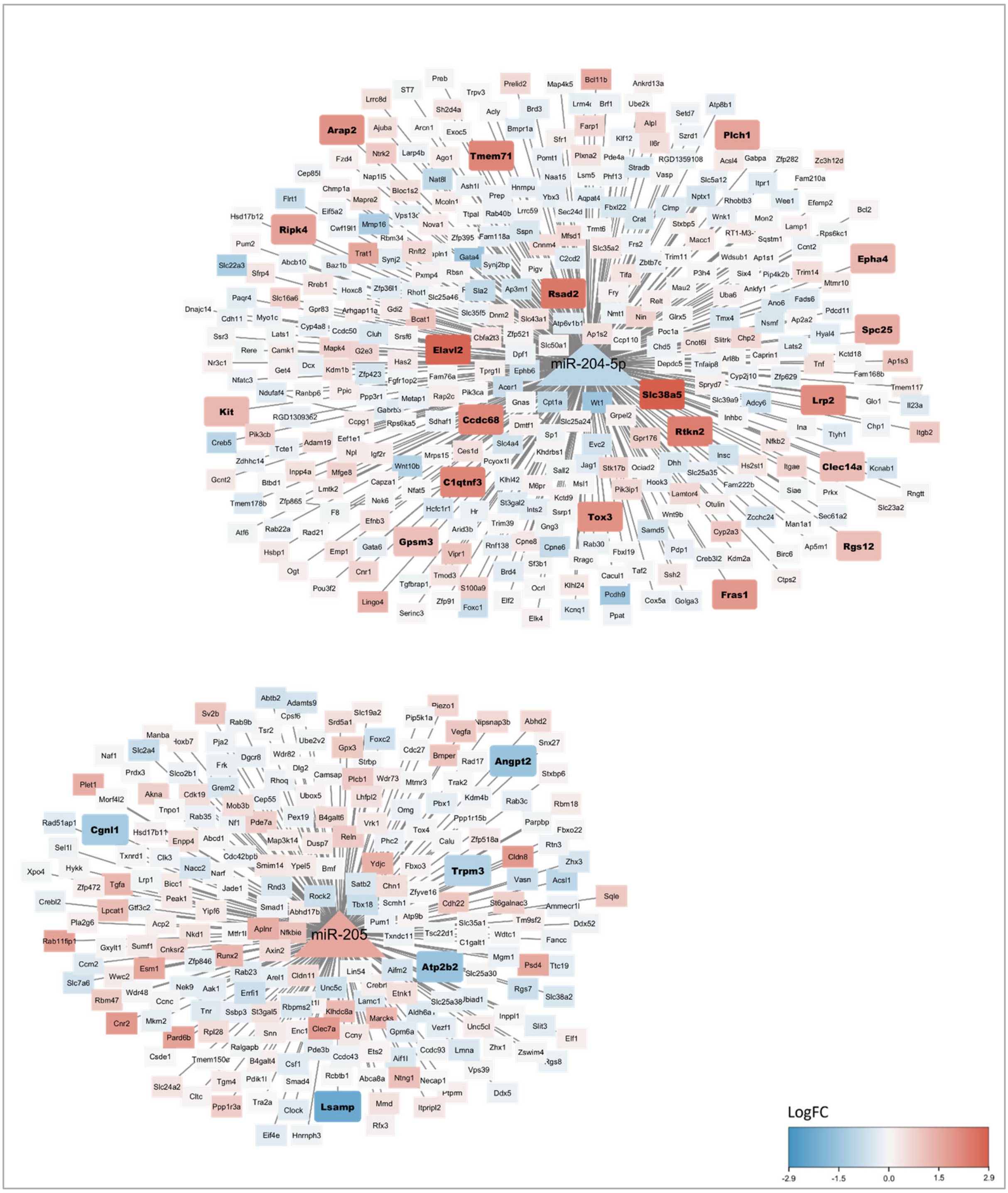

Figure S4. Clustered mRNA targets for miRNA candidates (miR-205 & miR-204-5p). miRNA/miR = micro-RNA, downstream rectangular targets represent mRNAs/messenger RNA. LogFC = Log2-fold change expression (colour gradient) for control vs HPH rats ((-2.9) – (2.9)). bold and enlarged mRNAs are differentially expressed (FDR *q*<0.05, log2FC>1). This network was generated using Cytoscape ver. 3.10.2.

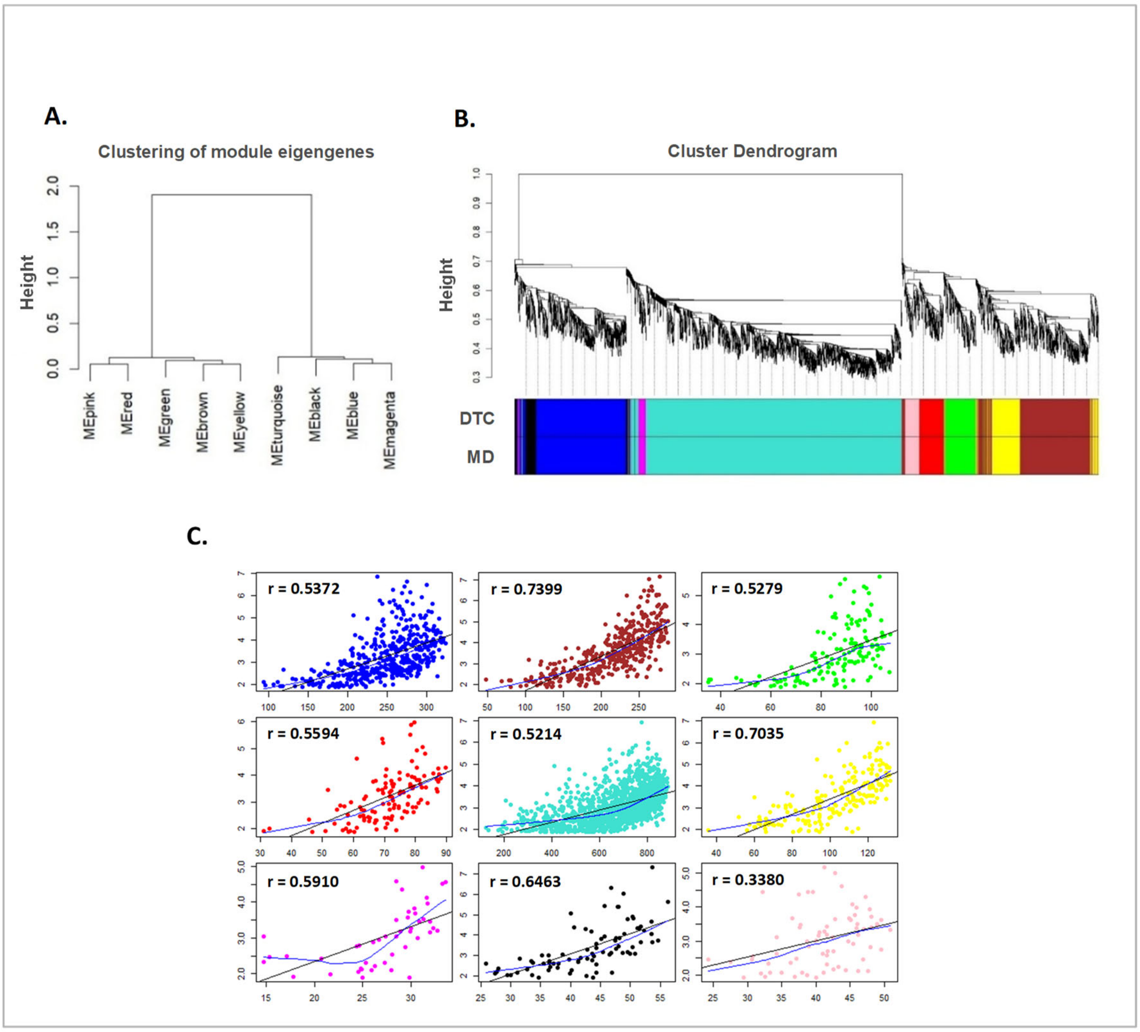

Figure S5. Weighted gene expression analysis (n=2762, FDR q<0.05, log2FC>0.5). **A**) Dendrogram representing grouped clustering of modules. **B**) Dendrogram representing grouped clustering of genes and module range for genes. **C**) Module separated scatter plots rendered against significance (y-axis) and intra-module connectivity (kWithin, x-axis). Greater fit to regression (Correlation(r) 0-1) = greater overall module connectivity. Weighted Gene Co-expression Analysis (WGCNA) ver. 1.73. Pearson⊗s correlation coefficient (PCC) employed to evaluate relationship between eigengenes and trait of interest (HPH, hypoxia induced pulmonary hypertension). Abbreviations: DTC, Dynamic Tree Cut; MD, Merged Dynamic; ME, module eigengene. Figure(s) generated using R. (Version 4.3.2).

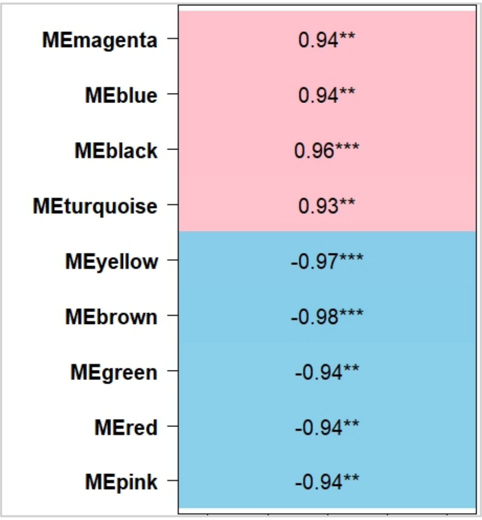

Figure S6. Binary module eigengene (ME) - trait association for all genes (HPH vs. Control, n= 2762, FDR q<0.05, log2FC>0.5). Generated using Pearson's correlation coefficient (PCC) to investigate relationship between hypoxia induced pulmonary hypertension (HPH) with module gene expression. ** p < 0.01; *** p <0.001. Figure generated using R. (Version 4.3.2).

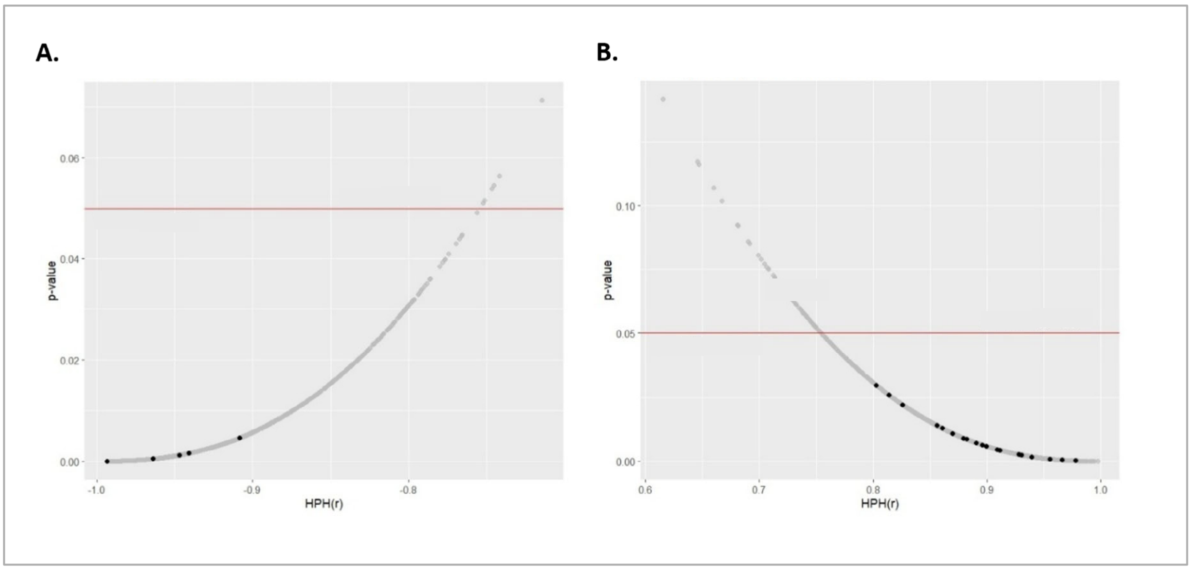

Figure S7. Binary module-trait association for all genes (HPH vs. Control, n= 2762, FDR q<0.05, log2FC > 0.5). A) Scatter plot, p value against persons coefficient (r) for all upregulated genes (n=1832, n(p<0.05) =1764, n n(p>0.05) =68). Statistically significant correlation (FDR p < 0.05). HPH(r)>0.6 = strong relation, HPH(r)>0.3 & <0.6 = moderate relation. Black points indicate predicted genes of downstream circCnot6l. B) Scatter plot, p value against persons coefficient for all downregulated genes (n =930, n(p<0.05) =924, n n(p>0.05) =6). p<0.05 indicates statistically significant correlation, HPH(r) > 0.6 = strong, 0.3 = moderate, black points indicate predicted genes of downstream circNtrk3. Figure(s) generated using R. (Version 4.3.2) and ggplot2 (Version 4.0.2).

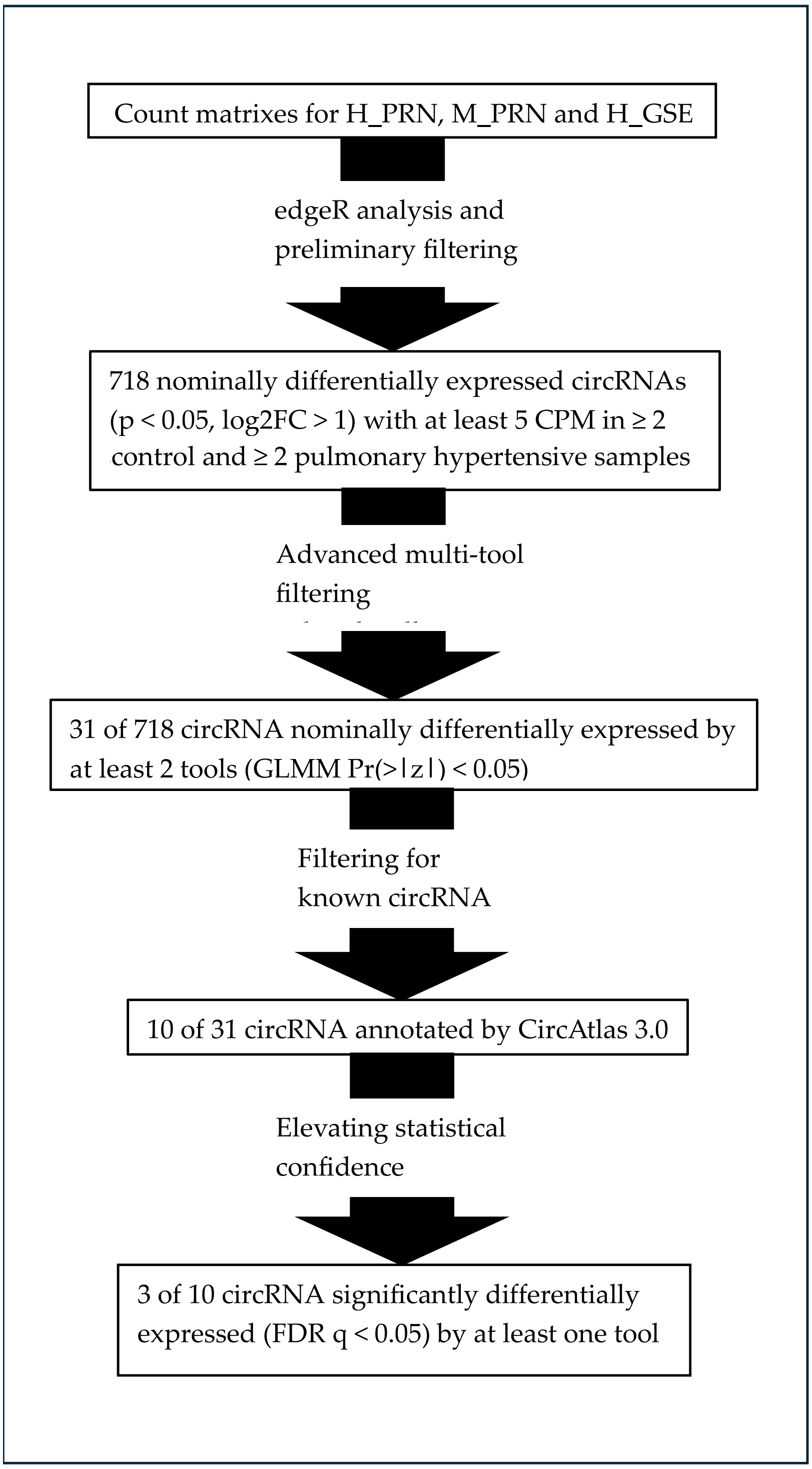

Figure S8. Step-by-step filtering to identify statistically significant and annotated circular RNA (circRNA) candidates. The three datasets used are PRJNA809145 (H_PRN), GSE159668 (H_GSE), and PRJNA732522 (M_PRN). A total of 718 circRNAs were nominally differentially expressed ($p < 0.05$, log2 fold change > 1) and demonstrated an acceptable level of statistical robustness (CPM > 5 and detected in ≥ 2 control and ≥ 2 pulmonary hypertensive samples) across the individual datasets. Among these 718 circRNAs, 31 were identified as nominally differentially expressed by at least two detection software programs and also exhibited concordant inter-sample expression (GLMM Pr(>|z|) < 0.05). Of these 31 circRNAs, 10 were annotated in CircAtlas 3.0. Among the 10 annotated circRNAs, three were significantly differentially expressed, with a Benjamini-Hochberg adjusted *p*-value (FDR *q*) < 0.05. Abbreviations: CPM, counts per million; FDR, false discovery rate; GLMM, generalized linear mixed modelling; log2FC, log2 fold-change.

Table S1. Top 10 upregulated biological processes associated with HPH

RANK	BIOLOGICAL FUNCTION	FDR Q-VALUE
1	positive regulation of cell migration	0.00438
2	regulation of wound healing	0.00491
3	cell adhesion mediated by integrin	0.00500
4	positive regulation of vasculature development	0.0131
5	positive regulation of hydrolase activity	0.0219
6	positive regulation of multicellular organismal process	0.0289
7	positive regulation of angiogenesis	0.0411
8	regulation of cellular process	0.0436
9	regulation of GTPase activity	0.0485
10	regulation of hemostasis	0.0499

Gene ontology for upregulated single rank biological processes filtered by pathogenesis (HPH) and significance (FDR q-value). Ontology was run using GOrilla enrichment webtool using default parameters (Single ranked) for all upregulated genes (n=2510. FDR $q < 0.05$).

Table S2. Top 10 downregulated biological processes associated with HPH

RANK	BIOLOGICAL FUNCTION	FDR Q-VALUE
1	supramolecular fiber organization	0.0089
2	anatomical structure development	0.0182
3	regulation of multicellular organismal development	0.0186
4	response to cytokine	0.0230
5	actin filament organization	0.0250
6	signal transduction	0.0255
7	negative regulation of blood vessel diameter (vasodilation)	0.101
8	vasoconstriction	0.109
9	response to abiotic stimulus	0.119
10	regulation of transmembrane transport	0.203

Gene ontology for downregulated single rank biological processes filtered by pathogenesis (HPH) and significance (FDR q-value). Ontology was run using GOrilla enrichment webtool using default parameters (Single ranked) for all downregulated genes (n=2040. FDR $q < 0.05$).

Table S3. Comparison of In Silico circRNA detection pipelines

Tool	*Last update*	*version*	*Tool type*	*Aligner*	*BSJ quantification*	*Programming language*
CIRI2	2021	2.1.1	*De novo*; annotation	BWA	YES	Perl
CIRIquant	2023	1.1.3	*De novo*; annotation	HISAT2	YES	Perl, Python
CircRNA_finder	2019	1.2	*De novo*	STAR	YES	Perl, AWK
find_circ	2015	1.2	*De novo*	Bowtie2	YES	Python

Detailed metadata for four independent software packages used to identify and or quantify circRNA. Abbreviations: BSJ, back splice junction; BWA, Burrows-Wheeler Aligner; HISAT2, Hierarchical Indexing for Spliced Alignment of Transcripts 2; STAR, Spliced Transcripts Alignment to a Reference.

Table S4. Threshold criteria for each stage of gene analysis

Input gene universe	*Log2FC threshold*	*FDR threshold*	*Number of genes*	*Purpose*
All statistically significant genes	No threshold	< 0.05	4,550 (2,100 upregulated and 2,040 downregulated in HPH vs. control)	For GOrilla single ranked gene ontology analysis
Statistically significant genes that show at least marginal change in expression	> 0.5 or < -0.5	< 0.05	2,762 (1,832 upregulated and 930 downregulated in HPH vs. control)	For WGCNA
Statistically significant genes that show at least 2-fold change in expression at 2-fold or greater change in expression	> 1 or < -1	< 0.05	1,335 (1,062 upregulated and 273 downregulated in HPH vs. control)	For target prediction analysis

Abbreviations: FDR, false discovery rate; GOrilla, Gene Ontology enRIchment anaLysis and visuaLizAtion tool; HPH, hypoxia-induced pulmonary hypertension; log2FC, log2 fold-change; WGCNA, Weighted gene co-expression network analysis.

Table S5. Sample level metadata for PRJNA809145/H_PRN

Sample	*Group assignment*	*Treatment*	*Sample accession*	*Tissue*	*Library type*	*Sequencing depth*	*Read length*
Control 1	Normoxia	21% O_2	SRX14246012	TPAT	RNA-Seq	58.2M	150bp
Control 2	Normoxia	21% O_2	SRX14246013	TPAT	RNA-Seq	60.5M	150bp
Control 3	Normoxia	21% O_2	SRX14246021	TPAT	RNA-Seq	52.0M	150bp
Control 4	Normoxia	21% O_2	SRX14246022	TPAT	RNA-Seq	62.5M	150bp
Experiment 1	Atmospheric hypoxia	10% O_2	SRX14246023	TPAT	RNA-Seq	63.6M	150bp
Experiment 2	Atmospheric hypoxia	10% O_2	SRX14246024	TPAT	RNA-Seq	72.8M	150bp
Experiment 3	Atmospheric hypoxia	10% O_2	SRX14246025	TPAT	RNA-Seq	43.6M	150bp

Abbreviations: bp, base pairs; M, million; O_2, oxygen; RNA-Seq, RNA-sequencing; TPAT, total pulmonary artery tissue.

Table S6. Sample level metadata for GSE159668/H_GSE

Sample	*Group assignment*	*Treatment*	*Sample accession*	*Tissue*	*Library type*	*Sequencing depth*	*Read length*
Control 1	Normoxia	21% O_2	SRX9317350	Lung	RNA-Seq	52.4M	151bp
Control 2	Normoxia	21% O_2	SRX9317351	Lung	RNA-Seq	53.3M	151bp
Control 3	Normoxia	21% O_2	SRX9317352	Lung	RNA-Seq	55.2M	151bp
Experiment 1	Atmospheric hypoxia	9.5-10.5% O_2	SRX9317353	Lung	RNA-Seq	55.3M	151bp
Experiment 2	Atmospheric hypoxia	9.5-10.5% O_2	SRX9317354	Lung	RNA-Seq	51.0M	151bp
Experiment 3	Atmospheric hypoxia	9.5-10.5% O_2	SRX9317355	Lung	RNA-Seq	49.3M	151bp

Abbreviations: bp, base pairs; M, million; O_2, oxygen; RNA-Seq, RNA-sequencing.

Table S7. Sample level metadata for PRJNA732522/M_PRN

Sample	*Group assignment*	*Treatment*	*Sample accession*	*Tissue*	*Library type*	*Sequencing depth*	*Read length*
Control 1	Healthy	Standard volume saline	**SRX11002198**	Lung	RNA-Seq	56.6M	150bp
Control 2	Healthy	Standard volume saline	SRX11002199	Lung	RNA-Seq	61.5M	150bp
Control 3	Healthy	Standard volume saline	SRX11002200	Lung	RNA-Seq	63.4M	150bp
Experiment 1	Monocrotaline induced hypoxia	single subcutaneous monocrotaline injection (60 mg/kg)	SRX11002201	Lung	RNA-Seq	62.8M	150bp
Experiment 2	Monocrotaline induced hypoxia	single subcutaneous monocrotaline injection (60 mg/kg)	SRX11002202	Lung	RNA-Seq	60.7M	150bp
Experiment 3	Monocrotaline Induced hypoxia	single subcutaneous monocrotaline injection (60 mg/kg)	SRX11002203	Lung	RNA-Seq	62.8M	150bp

Abbreviations: bp, base pairs; kg, kilogram; M, million; mg, milligram; O2, oxygen; RNA-Seq, RNA-sequencing.
